# Cobalt Toxicity Presenting as Bilateral Optic Neuropathy

**DOI:** 10.31486/toj.25.0025

**Published:** 2025

**Authors:** Fatima-tun-Nissa Raza, Can Kocasarac

**Affiliations:** ^1^ Department of Neuro-Ophthalmology, University of Pittsburgh Medical Center, Pittsburgh, PA; ^2^ Department of Ophthalmology, Duke University Medical Center, Durham, NC

**Keywords:** *Arthroplasty–replacement–shoulder*, *electroretinography*, *optic nerve*, *scotomas*, *toxic optic neuropathy*

## Abstract

**Background:**

Cobalt, a ferromagnetic metal with a wide range of uses, has been known to cause systemic toxicity in humans that results in thyroid dysfunction, cardiomyopathy, hematologic abnormalities, and neuropathy. Arthroprosthetic cobaltism is a term used to describe systemic cobalt toxicity originating from cobalt-containing prosthetic joints. Ocular toxicity from elevated serum levels of cobalt is a rare phenomenon.

**Case Report:**

We describe a case of bilateral toxic optic neuropathy resulting from a damaged cobalt-containing shoulder prosthesis in a 76-year-old female. The patient exhibited classic clinical findings of toxic optic neuropathy, with painless insidious vision loss, dyschromatopsia, and bilateral central scotomas. Clinical testing showed a progressive reduction in the thickness of the peripapillary retinal nerve fiber layer and ganglion cell complex on optical coherence tomography. Magnetic resonance imaging of the brain and orbits without contrast were unrevealing. Extensive laboratory testing ruled out infectious, inflammatory, demyelinating, and nutritional causes of optic neuropathy. However, slightly elevated levels of serum cobalt were found. Considering the possibility of arthroprosthetic cobaltism, arthrocentesis from the patient's shoulder prosthesis was performed and revealed that the cobalt levels in her synovial fluid were 35 times higher than the normal limit. Replacement of the damaged prosthetic joint resulted in a decrease in the patient's serum cobalt levels and improvement of her vision.

**Conclusion:**

While arthroprosthetic cobaltism from hip joints has been reported, to our knowledge, ours is the first case to prove a damaged shoulder prosthesis was the etiology. The patient's relatively low levels of serum cobalt and the lack of systemic features are also intriguing.

## INTRODUCTION

Cobalt, a ferromagnetic transition metal with diverse commercial, industrial, and military uses,^1^ is an essential trace element in the human body because it is a key component of vitamin B_12_ (cobalamin).^2^ Cobalt is important for cell mitosis, for forming some proteins necessary for myelin sheath synthesis, and for the synthesis of amino acids and some neurotransmitters.^2^ Leyssens et al described 4 sources of cobalt toxicity: occupational, environmental, dietary, and iatrogenic.^3^ Cobalt exposure in humans can occur through ingestion, respiration, dermal contact, and cobalt-containing biomaterials.^2^

Cobalt systemic toxicity can lead to cardiomyopathy, thyroiditis, polycythemia, peripheral neuropathy, and dysfunction of the optic and vestibulocochlear nerves.^3-5^ Similarly to nickel, cobalt can cause contact dermatitis.^3,4^ Inhalation of cobalt dust, which has been documented in factory workers who drill hard metal, can elicit hypersensitivity pneumonitis.^4^ Ocular toxicity can manifest as optic neuropathy, retinopathy, and chorioretinal degeneration.^4,6^ Extraocular movement abnormalities in the context of cobalt toxicity have also been described.^7^

We present a case of cobalt-induced toxic optic neuropathy originating from a damaged shoulder prosthesis and review the literature on cobalt ophthalmic toxicity.

## CASE REPORT

A 76-year-old female was referred to our clinic for evaluation of bilateral painless progressive vision loss that had begun approximately 1.5 years prior to presentation. She had multiple comorbidities including diabetes mellitus, hypertension, hyperlipidemia, chronic kidney disease, mixed sensorimotor polyneuropathy affecting both legs, rheumatoid arthritis, and gout. The patient had had multiple revisions of a left hip prosthesis and had a prosthetic right shoulder. Her family history was remarkable for retinal detachment in her mother and several cousins, but no other history of blindness was reported.

When we evaluated the patient in our clinic in January 2023, visual acuity was 20/125 in both eyes and did not improve on pinhole testing or refractive correction. The referring ophthalmologist's notes from December 2022 documented a visual acuity of 20/80 in the right eye and 20/70 in the left. On our examination, her pupils were brisk and symmetric in size and reactivity. Intraocular pressures were normal at 17 mm Hg and 18 mm Hg. Visual fields were full on confrontation testing, and extraocular movements were full and painless. Color vision was profoundly reduced in both eyes; the patient could read only a single Ishihara test plate with her right eye and none with the left. Slit lamp examination was remarkable for bilateral intraocular lens implantation. The posterior lens capsules in both eyes had undergone YAG laser capsulotomies to treat posterior capsular opacification. Fundus examination showed attenuation of arterioles, likely attributable to systemic hypertension. Optic discs had substantial peripapillary atrophy in both eyes but did not show any signs of pallor or edema. The cup-to-disc ratio was 0.3 in both eyes.

Neurologic evaluation revealed intact higher mental function and cranial nerve examination. Motor system examination was limited by the presence of right ankle and left hip braces but was otherwise normal. Sensations were symmetric to light touch but reduced to vibration in both upper extremities. Sensory examination in the lower limbs was limited by the presence of the ankle and hip braces. The patient was wheelchair-bound, so her gait could not be evaluated.

**Figure 1.**
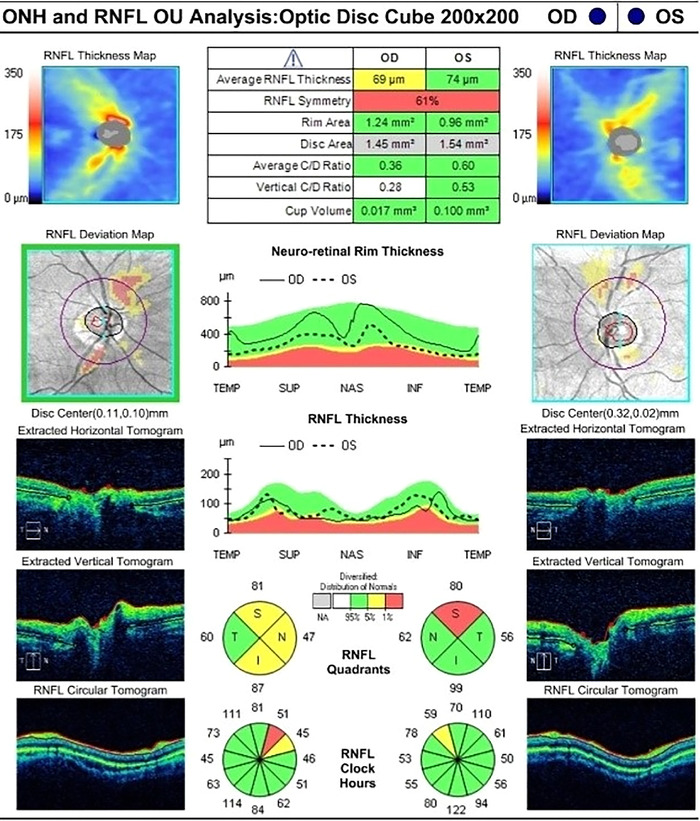
Initial optical coherence tomography showed borderline thinning of the peripapillary retinal nerve fiber layer in the right eye. In the left eye, thinning of the peripapillary nerve fiber layer was noted in the superior quadrant, but the average thickness was within the normal range.

**Figure 2.**
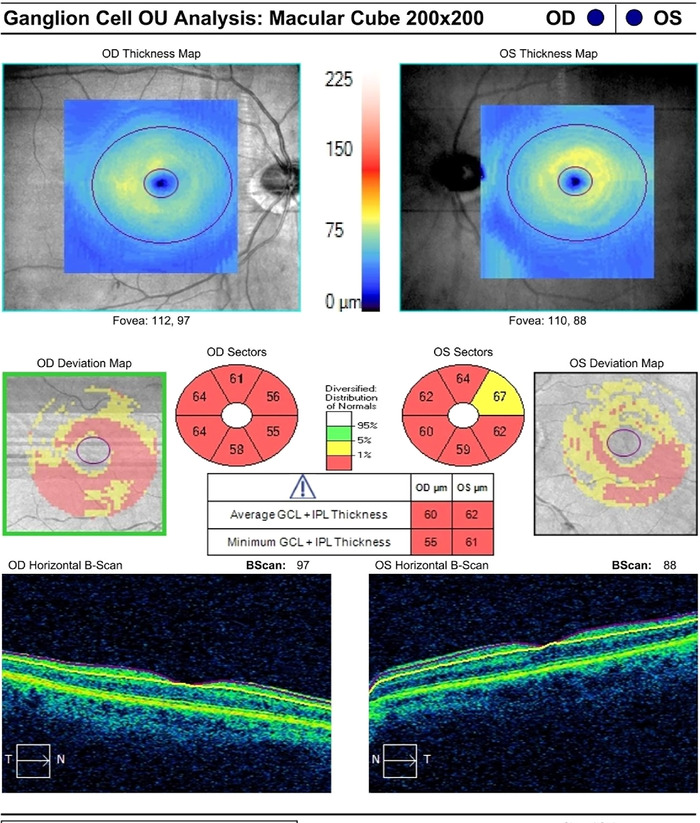
Initial ganglion cell and inner plexiform layer complex analysis showed diffuse thinning in both eyes.

Measurement of the thickness of the retinal nerve fiber layer via optical coherence tomography (OCT) showed borderline thinning of the right peripapillary retinal nerve fiber layer, sparing the temporal quadrant, with an average measurement of 69 μm (Figure 1). The left nerve had thinning of the retinal nerve fiber layer in the superior quadrant, but average thickness measured within the normal range at 74 μm. Ganglion cell complexes that contain the cell bodies of the neurons making up the optic nerve showed diffuse thinning in both eyes, measuring 60 and 62 μm. (Figure 2). OCT of the macular region, which includes the fovea, showed normal healthy maculae in both eyes.

**Figure 3.**
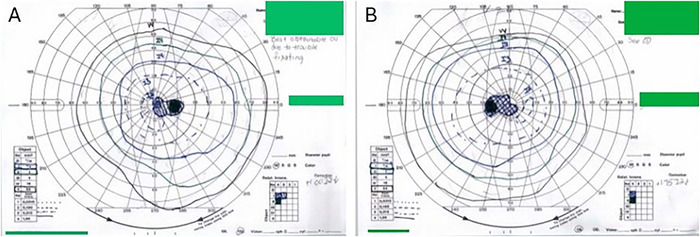
Initial Goldmann visual field test showed a central scotoma (A) in the left eye and (B) in the right eye.

Automated Humphrey visual field 30-2 testing was unreliable, and a neurologic pattern of field loss could not be demonstrated. Manual Goldmann visual field test showed bilateral central scotomas (Figure 3). The patient's chronic kidney disease precluded the use of gadolinium contrast for magnetic resonance imaging (MRI), but non-contrast-enhanced MRI of the brain showed atrophic brain changes, as well as nonspecific white matter lesions that were attributed to chronic small vessel disease. Visual electrophysiologic studies—visual evoked potentials (VEPs) and electroretinography (ERG)—were conducted to help localize the vision loss to either the retina or the afferent neurologic pathways, but these studies were confounded by the patient's drowsiness.

Laboratory testing was normal or negative for inflammatory, autoimmune, and infectious causes of optic neuropathy. Vitamin levels were all within range. The patient's medication list did not contain any agent suspicious for causing optic neuropathy. Heavy metal screen showed elevated levels of cobalt in the serum (5.5 μg/L; reference range, 0.1-0.4 μg/L) and in the urine (4.5 μg/L; reference range, <2.8 μg/L). The patient's drinking water was tested for heavy metals, and all results were normal.

Suspecting that one of her prostheses might be the source of the elevated cobalt levels, we contacted her orthopedic surgeon. The patient had been complaining of pain and stiffness in her right shoulder, so synovial fluid was aspirated from the joint and evaluated for heavy metals. Because elevated levels of cobalt were found in the synovial fluid (35 μg/L; reference range, <1 μg/L), the right shoulder joint prosthesis was determined to be the source of the cobalt toxicity.

At a follow-up visit in March 2023, the patient's visual acuity had deteriorated to 20/500 in both eyes, improving on pinhole testing to 20/150 in the right eye only.

She underwent revision of the right shoulder joint in July 2023. Intraoperative evaluation showed complete rupture of right rotator cuff tendons with a high-riding prosthetic humeral head. The patient had originally undergone a reverse total shoulder replacement with a Biomet comprehensive noncemented implant (Zimmer Biomet Holdings, Inc). The revision surgery was a long stem reverse total shoulder replacement. All metal and polyethylene components and cement were removed. Synovectomy of the right shoulder and tenotomy of the long head of the right biceps were performed, along with excision of the heterotopic bone of the right shoulder.

The patient reported subjective improvement in her vision at her postoperative orthopedic evaluation 2 months later. Neuro-ophthalmic evaluation performed 8 months after the surgery revealed that her visual acuity had improved to 20/30 in the right eye and 20/25 in the left. She could read 6 of 8 Ishihara test plates with the right eye and 7 of 8 plates with the left. Measurement of the retinal nerve fiber layer showed a slight decrease in both eyes. Visual fields were improved. Serum cobalt levels trended down, measuring 2.7 μg/L 2 months postoperatively and 1.7 μg/L 8 months after surgery.

## DISCUSSION

In 2010, Tower first used the term arthroprosthetic cobaltism to describe the phenomenon of systemic cobalt toxicity stemming from damaged prosthetic joints.^8^ The problem is increasingly being reported over time.^9^ To our knowledge, 16 cases documenting ocular cobalt toxicity originating from damaged prosthetic joints have been published. Tables 1 and 2 summarize the findings from these case reports.^4,6-8,10-21^

**Table 1.** Summary of the Systemic Features of the Previously Reported Cases of Arthroprosthetic Cobaltism with Ocular Involvement

Study	Age, years / Sex	Prerevision Implant Type	Postrevision Implant Type	Time to Onset of First Symptom	First Symptom	Co and Cr Serum Levels, μg/L	Systemic Findings
Steens et al, 2006^10^	53 / M	Ceramic on ceramic	Ceramic on metal	2 years	Hearing and vision loss	Co: 398 Cr: 56	Hearing loss, paresthesias, dermatitis
Rizzetti et al, 2009^11^	58 / F	Ceramic on ceramic	Metal on polyethylene	2 months	Hearing and vision loss	Co: 549 Cr: 54	Hearing loss, lower limb hyposthenia, hypothyroidism
Tower, 2010^8^	49 / M	Metal on metal	Nonmetal on metal	3 months	Axillary rashes	Co: 35-122 Cr: 28-63	Cognitive disturbances, hearing loss, dyspnea, diastolic dysfunction
Apel et al, 2013^12^	65 / M	Ceramic on ceramic	Ceramic on metal	NR	NR	Co: 355-446.4 Cr: 39-46	Malaise, cardiomyopathy, bulbar palsy, hypothyroidism
Ng et al, 2013^13^	39 / F	Metal on metal	NR (no intervention)	NR	NR	Co: 44.7 Cr: 30.9	Nausea, metallic taste
Dahms et al, 2014^14^	55 / M	Ceramic on ceramic to metal on polyethylene	Ceramic (unspecified)	1 year	NR	Co: 884 Cr: 49	Heart failure, hearing loss, hypothyroidism, fever of unknown origin, lymphadenopathy (mediastinal, left hip)
Harris et al, 2015^15^	57 / F	Ceramic on ceramic	Metal on polyethylene	NR	NR	Co: 788.1 Cr: 140	Hip pain, fatigue, memory loss, lower extremity sensory loss, tachycardia, hypothyroidism
Weber et al, 2015^18^	66 / F	Ceramic head	Metal on polyethylene	NR	NR	Co: 411 Cr: NR	Depression, fatigue, unintentional weight loss, paresthesias, disequilibrium, hearing loss, cardiomyopathy, polycythemia
Grillo et al, 2016^6^	66 / M	Ceramic on ceramic	Metal on metal	9 months	NR	Co: 1,078 Cr: NR	Hearing loss, abdominal distention, ankle swelling, left hip pain, diarrhea, rash, incoordination, cognitive difficulties
Peters et al, 2017^16^	71 / M	Ceramic on ceramic	Metal on polyethylene	6 months	Hip pain	Co: 596.5 Cr: 48.8	Abdominal pain, vomiting, diarrhea, hearing loss, vertigo, weight loss, hypothyroidism, cardiomyopathy
Ho et al, 2018^17^	63 / M	Ceramic on ceramic	NR	1 year	Painful paresthesias	Co: 280-779 Cr: NR	Hearing loss, painful paresthesias, cardiomyopathy
Lecoanet et al, 2019^20^	69 / F	Ceramic acetabular liner	Metal on polyethylene	<1 year	Fever, asthenia, tachycardia, weight loss, left groin pain	Co: 1,461 Cr: 65.9	Cognitive decline, behavioral disorders, memory decline, hearing loss, vision loss, asthenia, anorexia, weight loss, dysgeusia
Garcia et al, 2020^4^	59 / F	Ceramic on ceramic	Metal on polyethylene	2-3 months	Fatigue and hair loss	Co: >1,000 Cr: 36.9	Fatigue, hair loss, hearing loss, hypothyroidism, sinus tachycardia, headaches, numbness, paresthesias
Rattay et al, 2021^7^	53 / M	Ceramic head	Metal head	4 years	Hearing loss	Co: 85 Cr: 25	Hypesthesia/dysesthesia, paraparesis, unsteady gait, hair loss, unexpected weight loss, cardiomyopathy, polycythemia, hearing loss
Shaikh et al, 2022^21^	58 / F	Metal on metal	NR	1 year	Painless, progressive vision loss	Co: 734 Cr: NR	Bilateral hearing loss, tinnitus, unintentional weight loss, thyroid dysfunction, lower extremity tremor
Samargandi et al, 2024^19^	64 / F	Ceramic on ceramic	Metal on polyethylene	1 year	Sudden left-sided deafness	Co:86.3 Cr: 234.4	Deafness, polycythemia, fatigue, weakness, paresthesias, cardiomyopathy, hypothyroidism

Co, cobalt; Cr, chromium; F, female; M, male; NR, not reported.

**Table 2.** Summary of the Ophthalmic Features and Investigations of the Previously Reported Cases of Arthroprosthetic Cobaltism With Ocular Involvement

Study	Time to Onset of Visual Symptoms	Presenting Visual Acuity	Final Visual Acuity	Ocular Findings	Magnetic Resonance Imaging	Electroretinography	Visual Evoked Potentials
Steens et al, 2006^10^	2 years	Could recognize outlines and colors but could not read	Able to work on computer with strong glasses	Optic neuropathy and retinopathy OU	NR	NR	NR
Rizzetti et al, 2009^11^	2 months	NR	NR (partially improved)	Optic neuropathy OU	Enhancement of optic nerves and tracts	NR	Irregular cortical visual responses
Tower, 2010^8^	3 years	NR	NR (stable)	Optic neuropathy (eye NR)	NR	NR	NR
Apel et al, 2013^12^	4 years	20/200 OU	20/20 OD 20/30 OS	Retinopathy OU	Chronic ischemic changes in deep white matter	Persistent abnormalities in all phases suggesting inner retinal pathology OU	Normal
Ng et al, 2013^13^	2 years	20/16 OU	20/16 OU	Bilateral ocular discomfort, chorioretinal degeneration	NR	Normal	Well developed with normal latencies OU
Dahms et al, 2014^14^	1 year	NR	NR (slight recovery)	Vision loss (eye NR)	NR	NR	NR
Harris et al, 2015^15^	NR	NR	NR (improved)	Reduced visual acuity, subjective nonspecific visual changes (eye NR)	NR	NR	NR
Weber et al, 2015^18^	1 year	Count fingers OU	Count fingers OU	Optic neuropathy and retinopathy OU	Unremarkable	Severe cone dysfunction and low normal rod function	NR
Grillo et al, 2016^6^	9 months	20/80 OD (history of amblyopia) 20/30 OS	20/80 OD (history of amblyopia) 20/25 OS	Optic neuropathy	NR	Normal	Decreased central amplitudes
Peters et al, 2017^16^	NR	NR	NR	Nonspecific visual impairment (eye NR)	NR	NR	NR
Ho et al, 2018^17^	2 years	20/80 OD 20/200 OS	Count fingers OU	Optic neuropathy OU	Enhancement of optic nerves OU	NR	NR
Lecoanet et al, 2019^20^	<1 year	NR	NR (improved)	Optic neuropathy OU (disc edema)	Heavy metal accumulation in basal ganglia and caudate nucleus (hyperintensity on T1 and hypointensity on T2)	NR	NR
Garcia et al, 2020^4^	14 months	20/600 OD 20/800 OS	20/125 OU	Optic neuropathy and retinopathy OU	Normal	mfERG: Decreased tracing amplitudes OU	NR
Rattay et al, 2021^7^	5 years	20/200 OU	20/60 OU	Abduction deficits OU (without diplopia), vertical saccade slowing, saccadic pursuit movements, delayed direct and indirect pupillary response to light suggesting optic neuropathy	NR	NR	NR
Shaikh et al, 2022^21^	1 year	Baseline: Count fingers OD (amblyopia) 20/30 OS Presenting: Count fingers OU	Count fingers centrally, with improved colors and fields OU	Preexisting myopic degeneration OD, optic neuropathy and retinopathy OU	Perineural enhancement of optic nerves OU, enhancement of the right cochlea	Full-field ERG OS: Mixed a- and b-wave amplitudes, depressed photopic b-wave with a smaller photopic a-wave, depressed flicker ERG	NR
Samargandi et al, 2024^19^	1 year	NR	NR (improved)	Optic neuropathy OU	Normal	NR	Abnormal

ERG, electroretinography; mfERG, multifocal electroretinography; NR, not reported; OD, right eye (oculus dexter); OS, left eye (oculus sinister); OU, both eyes (oculus uterque).

Males and females were affected equally (8:8), and patient ages ranged from 39 to 71 years. Visual acuity data were not reported in 8 cases.^8,10,14-16,19,20^ Of the cases that reported objectively measured visual acuity, improvement in vision was documented in 4 cases.^4,6,7,12^ One case reported worsening vision,^17^ and 3 cases reported stability.^13,18,21^

Apel et al described retinopathy only.^12^ Ng et al reported chorioretinal degeneration.^13^ Optic neuropathy without retinopathy was described in 7 reports,^6-8,11,17,19,20^ while 4 cases described concurrent optic neuropathy and retinopathy.^4,10,18,21^ Rattay et al described extraocular movement abnormalities.^7^

Serum cobalt levels in the patients in these studies ranged from 35 μg/L^8^ to 1,461 μg/L.^20^ At present, US Food and Drug Administration guidelines do not specify cutoff levels of cobalt in the blood for detection of arthroprosthetic cobaltism, citing a lack of evidence.^22^ The Medicines & Healthcare products Regulatory Agency (MHRA) in the United Kingdom suggests that whole blood cobalt levels ≥7 ppb (equivalent to 119 nmol/L or 7 μg/L) may indicate wear or corrosion of the joints. The MHRA also suggests that this threshold may be lower for stemmed total hip replacements.^23^ A serum cobalt level of 0.19 μg/L is considered normal in the general population.^5,24^ Concentrations ≥1.0 μg/L indicate possible environmental or occupational exposure.^25^

ERG is a diagnostic test that measures the electrical responses of the retina to light stimulation. Six of the case reports included ERG results.^4,6,12,13,18,21^ Garcia et al reported that multifocal ERG showed decreased tracing amplitudes in both eyes.^4^ Grillo et al and Ng et al reported normal ERG.^6,13^ ERG studies in the case described by Apel et al suggested an inner retinal pathology with persistent abnormalities in all phases of ERG.^12^ Weber et al reported that full-field ERG showed severe cone dysfunction.^18^ Shaikh et al reported depressed photopic b-wave and flicker ERG amplitudes.^21^

VEPs are electrical signals generated in the brain in response to a visual stimulus and can be measured using electrodes placed on the scalp. VEP results were described in 5 cases.^6,11-13,19^ Grillo et al described multifocal VEP with decreased central amplitudes.^6^ Rizzetti et al described irregular cortical visual responses.^11^ VEP was normal in the cases reported by Apel et al^12^ and Ng et al.^13^ Samargandi et al reported an abnormal VEP but did not describe the abnormality.^19^

Rizzetti et al^11^ and Ho et al^17^ described contrast enhancement of the optic nerves on MRI. Lecoanet et al reported T1 hyperintensity and T2 hypointensity in the basal ganglia and caudate on MRI, corresponding to cobalt deposition.^20^ Shaikh et al described perineural enhancement of the optic nerves after contrast administration.^21^ We did not administer contrast to our patient because of her poor renal function.

To our knowledge, this case is the first report of ocular cobalt toxicity associated with a shoulder prosthesis. The cases summarized in Tables 1 and 2 describe ocular cobalt toxicity related to hip prostheses. Khan et al reported a case of cutaneous titanium metallosis caused by a damaged shoulder prosthesis,^26^ but in our review of the literature, we could not find any cases of systemic heavy metal toxicity associated with shoulder prostheses.

Studies have shown that patients who underwent shoulder joint replacement have lower whole blood levels of cobalt than patients who underwent hip replacement.^27^ Consistent with this pattern, the serum cobalt concentration in our patient was lower than the levels reported in the other studies. However, there is conflicting evidence about the relationship between systemic cobalt levels and the occurrence of symptoms.^28^ Finley et al concluded that the earliest symptoms of toxicity were noted at blood cobalt levels >300 μg/L.^29^ A review by Gessner et al found that 17% of the reported cases of arthroprosthetic cobaltism had blood cobalt levels <20 μg/L.^9^ Despite this inconsistency, the risk of whether an individual will develop systemic toxicity depends on multiple factors that determine susceptibility, such as disease states and genetic polymorphisms.^5,30^

Metal ions in the blood can exist in bound and unbound forms. In bound form, metal ions are less bioavailable; hence, the toxicologically active form of cobalt is free divalent Co (II) ions.^5,31^ Strong cobalt binding sites in the serum include albumin, transferrin, and reduced glutathione.^5,30^ Conditions that can alter the binding of the cobalt ions to these molecules—such as end-stage renal disease, genetic polymorphisms, hypercholesterolemia, and hypoalbuminemia—can affect how much free cobalt is available in the blood.^5,30^ Our patient had renal failure, which is associated with higher than normal levels of ischemia-modified albumin (a form of albumin that has a modified N-terminus and thus reduced cobalt binding capacity), as well as decreased cobalt clearance.^5,32^ Both of these factors should lead to elevated levels of free cobalt. Despite these factors, however, the serum cobalt level in our patient was not very high.

In their review of cobalt toxicity, Paustenbach et al report that the earliest effects of systemic cobalt toxicity are thyroid dysfunction and defective erythropoiesis, with symptoms occurring when the concentration of cobalt in the blood is >300 μg/L. Cardiac and neurologic effects are seen at much higher concentrations of >700 μg/L.^5^

Thyroid function and complete blood count results were normal in our patient. However, she may have had relative polycythemia, as anemia would be expected in the setting of poor renal function. Our patient had noteworthy peripheral neuropathy; however, she also had diabetes, and her neurology team did not definitively ascribe a cause to the neuropathy. Some unknown factors may have made her susceptible to the toxic effects of cobalt, even at low serum concentrations. Catalani et al suggested that neurologic symptoms associated with cobalt toxicity may be a form of mitochondrial neuropathy resulting from cobalt-induced mitochondrial injury.^30^ Unknown mitochondrial genetic polymorphisms could have predisposed our patient to an enhanced sensitivity to cobalt.

Even though our patient's clinical setting was atypical for cobalt toxicity, all evidence points to cobalt toxicity as the cause of her optic neuropathy because visual acuity, color vision, and visual fields improved after her serum cobalt levels were managed. The damaged shoulder prosthesis was clearly the source of the cobalt ions because the cobalt levels in the synovial fluid were 35 times higher than normal, and the patient's serum cobalt concentration began to trend downward after the surgery.

## CONCLUSION

This case report highlights our limited knowledge of the toxicokinetics of cobalt, demonstrates that cobalt toxicity is possible at low serum concentrations, and shows the potential for cobalt toxicity following shoulder prosthesis implantation. Ophthalmologists should have a low index of suspicion in the appropriate clinical setting. Our case also shows that liaising with the entire care team is important because the orthopedic surgeon initially suspected the shoulder joint as the culprit, performed the arthrocentesis to confirm his suspicions, and then conducted the corrective surgery that resulted in the patient regaining her vision.

## Notes

*This article meets the Accreditation Council for Graduate Medical Education and the American Board of Medical Specialties Maintenance of Certification competencies for Patient Care and Medical Knowledge.*
